# Does Restricted Ankle Joint Mobility Influence Hamstring Muscle Strength, Work and Power in Football Players after ACL Reconstruction and Non-Injured Players?

**DOI:** 10.3390/jcm12196330

**Published:** 2023-10-01

**Authors:** Łukasz Oleksy, Anna Mika, Maciej Kuchciak, Grzegorz Bril, Renata Kielnar, Olga Adamska, Paweł Wolański, Michał Deszczyński

**Affiliations:** 1 Faculty of Health Sciences, Department of Physiotherapy, Jagiellonian University Medical College, 31-008 Kraków, Poland; loleksy@oleksy-fizjoterapia.pl; 2 Oleksy Medical & Sport Sciences, 37-100 Łańcut, Poland; 3 Institute of Clinical Rehabilitation, University of Physical Education in Kraków, 31-571 Kraków, Poland; 4 Department of Physical Education, University of Rzeszów, 35-959 Rzeszów, Poland; mkuchciak@ur.edu.pl; 5 Physiotherapy and Sports Centre, Rzeszów University of Technology, 35-959 Rzeszów, Poland; g.bril@prz.edu.pl; 6 Institute of Health Sciences, Medical College of Rzeszów University, 35-315 Rzeszów, Poland; kielnarrenata@o2.pl; 7 Department of Orthopaedics and Rehabilitation, Medical Faculty, Medical University of Warsaw, 02-091 Warsaw, Poland; olgaadam98@gmail.com (O.A.); jm.deszczynski@me.com (M.D.); 8 Department of Physiology, Gdańsk University of Physical Education and Sport, 80-336 Gdańsk, Poland; pawel.wolanski@awf.gda.pl

**Keywords:** ankle dorsiflexion, football, hamstring muscles, strength, injury, WBLT

## Abstract

This study was aimed at observing how the limitation of ankle dorsiflexion ROM affects hamstring muscle Peak Torque/BW (%), Average Power (W), and Total Work (J), and whether this effect is similar in football players after ACL rupture and reconstruction and in those without injuries. The study included 47 professional football players who were divided into two groups: Group 1 (*n* = 24) after ACL reconstruction and Group 2 (*n* = 23) without injuries in the past 3 years. Based on the Weight-Bearing Lunge Test (WBLT), the following subgroups in Groups 1 and 2 were distinguished: N (normal ankle joint dorsiflexion) and R (restricted ankle joint dorsiflexion). The concentric isokinetic test (10 knee flexions and extensions at 60°/s) was performed on both limbs. Significantly lower values of Peak Torque/BW and Average Power were observed in Group 1 compared to Group 2, as well as in subjects with normal and restricted ankle dorsiflexion. However, no significant differences were noted for either group in any of the strength variables comparing subjects with normal and restricted ankle dorsiflexion. A poor and non-significant correlation was exhibited between the ankle joint range of dorsiflexion and all the strength variables. The area under the ROC curve (AUC) for all the evaluated variables in both groups was below 0.5, or very close to this value, indicating that ankle dorsiflexion ROM has no diagnostic accuracy for hamstring muscle strength. Based on the obtained results, it can be assumed that ankle dorsiflexion limitation, which is common in football players, is not a factor in weakening hamstring muscle strength, either in football players after ACL reconstruction or among those without injuries. However, some authors have reported that limited mobility of the ankle joint can have a destructive effect on the work of the lower limbs and may also be a factor in increasing the risk of football injuries in this area. Therefore, we have suggested that hamstring muscle weakness and increased risk of injury may occur due to factors other than limited ankle mobility. These observations may be of great importance in the selection of prevention methods by including a broad spectrum of physical techniques, not just exercises that focus on the improvement of mobility or stability of the lower limbs.

## 1. Introduction

Hamstring injuries have been identified as the most common injury experienced in football, occurring mainly during sprinting and high-speed running [1,2,3]. Numerous potential risk factors for hamstring injuries have been reported, such as decreased flexibility, hamstring muscle weakness, age, past injury history, fatigue and poor warm-up technique [2,4,5]. Some authors have also found that a decreased ankle dorsiflexion range of motion may represent a risk factor for hamstring injuries [3]. Moreover, several other lower limb injuries, i.e., ACL ruptures and Achilles and patellar tendon overuse, have also been associated with restricted ankle dorsiflexion range of motion (ROM) [6,7].

Previous studies indicated that restricted ankle dorsiflexion ROM significantly increases injury risk by modifying lower limb stiffness and landing forces [6,7]. It can potentially affect an athlete’s performance in multidirectional running and in unilateral dynamic balance [8], which are fundamental components of football [9]. In professional football, hamstring injuries most often occur during sharp turns or cutting, or when running at full speed, one-third occur during training, and the remainder occur during matches [10,11]. It was reported that, during sprinting, decreased ankle mobility may change the touchdown position of the foot, reducing the horizontal force production [12], and may lead to increased work required from the hamstring muscle, predisposing it to injury [13].

The integrated concept of kinetic chains indicates that muscle pathways form a large network of myofascial chains, transferring force between components, while the impairment of one joint may induce injury in others [14,15,16]. It has also been reported that longitudinal exposure to high-intensity, eccentric muscle actions, such as rapid acceleration, deceleration, jumping, and landing tasks, may increase the stiffness of the muscles and tendons [17], leading to decrease in the joint ROM [8,18]. It was further reported that muscles, which are chronically damaged by indirect trauma are more susceptible to contractures and muscular straining [14,15,16]. One of the major reasons for hamstring injuries is athletes returning to sport before making a complete recovery [19]. But the restrictions remaining in joint mobility and motor control after injury are an important but still underestimated factor in prevention strategies.

Moreno-Perez et al. [3] have suggested that the significant involvement of such high-intensity eccentric muscle actions during training and matches could lead to a reduction in ankle dorsiflexion ROM. The progressive decrease in ankle dorsiflexion ROM throughout a season was observed in 30% of all players [6].

Gabbe et al. [4] found that restricted ankle dorsiflexion was associated with the risk of hamstring injuries. On the other hand,, Van Dyk et al. [2] identified deficits in ankle dorsiflexion ROM as weak risk factors for hamstring injuries. They concluded that ankle dorsiflexion measurement has little clinical value in hamstring injury prediction. Thus, the relationship between ankle mobility and the risk of hamstring injuries is still poorly understood.

In previous studies, it has been suggested that hamstring muscle weakness [20] and reduced ROM at the ankle joint [4] are important risk factors of hamstring muscle strain injury. As reported by Kim et al. [21], in patients with an ACL rupture, a strength decrease was noted in both the quadriceps and hamstring muscles, with a significantly higher decrease in the quadriceps [22]. It is also not clear whether the limitation of ankle dorsiflexion common among footballers is more pronounced in this group, or if footballers with limited ankle mobility, having previously undergone ACL reconstruction also have weaker hamstring muscles than footballers with normal ankle mobility. It has not been clarified so far whether the limited mobility of the ankle joint may be a factor in weakening the hamstring muscles, and thereby potentially increasing the risk of their injury.

Although hamstring injuries are common in footballers, there are a limited number of studies presenting preventive protocols. Biz et al. [5], in a review paper, analyzed the physiotherapy protocols and specific exercises in professional and semi-professional football players. They concluded that the most common prevention protocols included exercises without any associated physical or manual therapy techniques. Therefore, there is a need for research assessing the impact of limited joint mobility on the risk of hamstring injury, which may be of great importance in the selection of prevention methods by including physical techniques.

This study was aimed at observing how the limitation of ankle dorsiflexion ROM affects hamstring muscle Peak Torque/BW (%), Average Power (W), and Total Work (J). We also sought to check whether this effect is similar in football players after ACL reconstruction and in those without injuries.

## 2. Materials and Methods

### 2.1. Participants

This study included football players from professional, regional teams (Table 1). They were divided into 2 groups:

Group 1 (*n* = 24)—football players after ACL reconstruction who had previously passed the return-to-sport tests (RTS) and were cleared to play. They were active players in their clubs and performed normal football training (injured leg—after ACL reconstruction, uninjured leg—contralateral limb without ACL injury).

Group 2 (*n* = 23)—football players without injuries within the past 3 years (both limbs were considered as equivalent because the initial statistical analysis did not show any significant differences between them; therefore, we examined the left limb equivalent of the involved limb and right limb equivalent of the uninvolved limb).

The inclusion criteria for subjects following ACL reconstruction were regular football training; first unilateral ACL rupture and reconstruction 2–3 years prior to the study; no additional injuries to the contralateral leg; age between 20 and 30 years; and normal BMI. The exclusion criteria were bilateral ACL reconstruction; graft rupture; ACL rupture without reconstruction; and serious injury in the contralateral leg. The inclusion criteria in Group 2 were a lack of any lower- or upper-limb or trunk injuries within the previous 3 years; age between 20 and 30 years; and normal BMI.

All football players were informed about the research protocol and provided their written informed consent to participate in the study. The approval of the Ethical Committee at the Regional Medical Chamber in Kraków was obtained for this research (23/KBL/OIL/2020). All procedures were performed in accordance with the 1964 Declaration of Helsinki and its later amendments.

**Figure 1:**
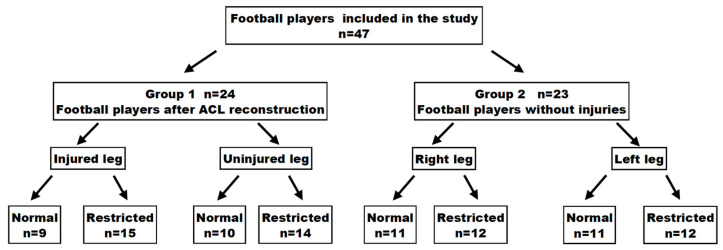
Study protocol.

Based on the previously reported cut-off value of ankle joint dorsiflexion (12 cm in the Weight-Bearing Lunge Test (WBLT), which is considered the norm) [23,24], the following subgroups in Groups 1 and 2 were distinguished (Figure 1):

- N (normal)—participants with a normal range of dorsiflexion in the ankle joint (12 cm or more);
- R (restricted)—participants with a reduced range of dorsiflexion in the ankle joint (below 12 cm).

### 2.2. Procedures

#### 2.2.1. Measurement of Ankle Joint Dorsiflexion Using the Weight-Bearing Lunge Test (WBLT)

A measuring tape (cm) was placed on the floor, the starting point (0 cm) aligned with the bottom corner of the wall. The players were instructed to stand facing the wall on their front lower limb, with 10 cm between the wall and the tips of the toes. The back lower limb was positioned by the subjects in such a way that they could stand in a stable and comfortable position. The subjects were allowed to hold onto the wall for balance during the test. In this position, the players lunged forward so that the knee touched the wall without taking their heel off the ground. If the heel lifted, the distance to the wall was shortened until the heel did not lift. If the heel did not rise, the distance was gradually increased until the heel rose, and the final distance at which the heel did not lift was marked [24,25]. The final distance between the tips of the toes and the wall was measured in cm; therefore, a longer distance indicated higher ankle joint dorsiflexion. Two repetitions of the WBLT were performed, and the higher score was analyzed. It was reported that the WBLT presented excellent intra-rater (ICC = 0.99) and inter-rater (ICC = 0.98) reliability [26,27].

#### 2.2.2. Isokinetic Test

Measurements were performed using an isokinetic dynamometer (System 4, Biodex Medical Systems, Shirley, New York, NY, USA) in a seated position, with the lower limb flexed in the hip joint to 90° and the knee axis of rotation concordant with the anatomical axis of the joint. The total range of motion (ROM) was set from full extension to full flexion of the knee joint. The movable arm of the dynamometer was fixed at 1/3 of the distal end of the tibia. Isokinetic testing in the concentric mode at an angular velocity of 60°/s was performed on both legs. The tests consisted of 10 flexions and extensions in the knee joint. The following hamstring muscle variables were analyzed: Peak Torque/BW (%); Average Power (W); and Total Work (J). The result was the mean value of 10 contractions. The reported reliability of the isokinetic test for knee flexion was good ICC = 0.88–0.97 [28,29].

#### 2.2.3. Statistical Analysis

STATISTICA 13.0 Pl software (StatSoft Poland, Krakow, Poland) was used. Data normality was tested with the Shapiro–Wilk test. The differences in muscle force variables between the limbs were tested with the paired t-test. Pearson’s correlation coefficient (*r*) between the WBLT value and strength variables was calculated (below 0.50—“poor,” between 0.50 and 0.75—“moderate”; between 0.75 and 0.90—“good”; above 0.90—”excellent”). The MANOVA test was implemented to establish the significance of differences in the strength variables across two independent factors (study group × WBLT (N or R)). The Cohen’s *d* effect size (ES) was calculated and interpreted as small (0.2–0.3), medium (0.5), or large (>0.8). Differences were statistically significant at a level of (*p* < 0.05).

The sensitivity, specificity, ROC (receiver operator characteristics) curve, and AUC (area under the curve) were calculated. ROC curves plot the true-positive rate (sensitivity) against the false-positive rate (1 minus the specificity) for the possible cut-off score. The AUC may be interpreted as the probability of restriction presence in ankle joint mobility correctly identifying potential hamstring muscle weakness in a player from randomly selected pairs of players who have a normal diminishment of their hamstring muscle strength. The AUC can range from 0.5 (no diagnostic accuracy) to 1.0 (perfect diagnostic accuracy).

## 3. Results

### 3.1. Strength Variables for Injured (Group 1) and Right (Group 2) Leg

Significantly lower values of Peak Torque/BW and Average Power were observed in Group 1 compared to Group 2, as well as in subjects with normal, as with restricted, ankle dorsiflexion (Table 2). However, no significant differences were noted in either group for any strength variables between subjects with normal or restricted ankle dorsiflexion (Table 2).

### 3.2. Strength Variables for Uninjured (Group 1) and Left (Group 2) Leg

Significantly lower values of Peak Torque/BW were observed in Group 1 compared to Group 2, as well in subjects with normal and with restricted ankle dorsiflexion (Table 3). The lower values of Average Power in Group 1 compared to Group 2 was demonstrated only in subjects with restricted ankle dorsiflexion (Table 3). However, no significant differences were noted in either group for any of the strength variables between subjects with normal or restricted ankle dorsiflexion (Table 3).

### 3.3. Correlations

A poor and non-significant correlation was observed between ankle joint range of dorsiflexion and all the strength variables (Table 4).

### 3.4. Diagnostic Value of Data

The area under the ROC curve (AUC) for all the evaluated variables in both groups was below 0.5, or very close to this value, indicating that the ankle dorsiflexion ROM has no diagnostic accuracy for hamstring muscle strength (Table 5).

## 4. Discussion

Football players post ACL reconstruction demonstrated significantly lower values of hamstring muscle strength and power compared to those without injuries. However, in both groups, no significant differences were noted in hamstring muscle strength between players with restricted and those with a normal range of ankle dorsiflexion. Footballers with a restricted range of ankle dorsiflexion presented similar hamstring muscle strength to those with a normal range. Also, the lack of correlation between hamstring strength and ankle dorsiflexion, as well as the low AUC value, indicate no effect of ankle joint mobility limitation on hamstring muscle strength. It is probable that the limited mobility of the ankle joint is not a factor in hamstring muscle weakness, regardless of whether the athlete has undergone ACL reconstruction or not. The amount of ankle joint mobility limitation appears to be similar in footballers after reconstruction and in healthy ones.

Football players often sustain ankle injuries, such as sprains, ligament reconstruction, or chronic instability, which lead to restrictions in ankle mobility with reduced dorsiflexion ROM [6]. Nonetheless, it has been reported that daily activities, i.e., walking or descending stairs, requires 10° of ankle dorsiflexion ROM, while sprinting or running require 20° to 30° [26]. Many authors have stated that restricted ankle dorsiflexion has a destructive effect on lower limb performance and increases the risk of injury [7,8]. Therefore, this problem may be especially present among footballers, who are a group of athletes particularly vulnerable to chronic injuries and overloading of the ankle joint. However, the potential influence of ankle mobility limitation on athletes’ performance and injury risk has been studied by many authors [1,4,7,8], and the obtained results are still equivocal.

In some studies, it has been shown that limited mobility of the ankle joint can have a destructive effect on the work of the lower limb’s entire kinematic chain and may also be a factor increasing the risk of football injuries in this area [30,31], especially those of the hamstring muscles [4]. In certain studies, it has been indicated that altered movement patterns and greater forces may predispose athletes to tissue overload [7,13]. Additionally, Almansoof et al. [15] have shown that the limited mobility of the ankle negatively affects the work of the calf. Based on the concept of myofascial chains and the transfer of forces through interconnected structures, the limited mobility of the ankle joint may disturb the work of the calf muscles [15,16]. Due to the fact that both the hamstrings and the gastrocnemius muscles are elements of the posterior muscle chain, it was suggested that disturbances in the transfer of forces through the calf affect the functioning of the hamstring muscles [16].

There are a number of theories suggesting an association between ankle dorsiflexion restriction and lower limb injury. It has been proposed that reduced ankle dorsiflexion may restrict the ability to pass the leg forwards over the foot and to lower the center of mass during squat-type movements [32]. It may also lead to abnormal lower-extremity biomechanics during closed-chain strengthening exercises [33] and also increase the risk of injury by altering lower-extremity stiffness and landing forces [7,34]. It has also been underlined that ankle dorsiflexion ROM has a crucial influence on performance in multidirectional sports movements [9,35] where altered proprioception or neuromuscular control can impact hamstring function and timing during the terminal phase of swing during sprinting, increasing the likelihood of hamstring injury at this time [6].

However, some authors have indicated a potential negative impact of foot dorsiflexion limitation on the hamstring muscle performance [4,20], while others have not confirmed such a relationship [36]. Furthermore, there are studies in which it is directly shown that footballers with limited ankle mobility do not suffer from hamstring injuries more often than footballers with normal mobility [2].

Bennell et al. [36] have found that the ankle dorsiflexion range was not a significant predictor of hamstring injury risk. Additionally, van Dyk et al. [2] have confirmed that the ankle dorsiflexion range of motion was a weak risk factor for hamstring injury. In their study, the differences between the injured and uninjured players were non-significant, with small effect sizes (d\0.2). Moreover, the ROC curve analyses showed an area under the curve of 0.61 for ankle dorsiflexion, indicating the poor combined sensitivity and specificity of these variables [2]. Our results seem to confirm van Dyk’s observation that limited ankle dorsiflexion is not a factor in hamstring muscle weakness and should not be considered a cause of hamstring injury. It is probable that the large number of hamstring muscle injuries experienced by football players has causes other than impaired mobility of the ankle joint.

In various studies, it is clearly indicated that ACL reconstruction in football players causes long-term deficits in both lower-limb muscle strength and joint mobility. Following ACL reconstruction, footballers are weaker than those without such an injury, which has been indicated by other authors [21,22] and in our research [37]. In this study, significantly lower hamstring strength and power were also observed in both limbs of football players after ACL reconstruction. We therefore hypothesized that the potential hamstring-impairing effect of limited ankle mobility may be greater in these players than in uninjured individuals. However, the results of this study did not confirm our hypothesis, indicating no significant relationship between the mobility of the ankle joint and the strength of the hamstrings in either group. The lack of correlation between the hamstring strength and ankle dorsiflexion, as well as the low AUC value reported in our study, indicates no effect of ankle joint mobility limitation on hamstring muscle strength, regardless of whether the athlete has undergone ACL reconstruction or not. The amount of ankle joint mobility limitation was similar in footballers post ACL reconstruction and in healthy ones.

There are some limitations of the study. The study is cross-sectional; therefore, no causal inferences can be concluded. Thus, the longitudinal monitoring of muscle strength and ankle dorsiflexion ROM would be of interest.

## 5. Conclusions

The hamstring muscles in football players following ACL reconstruction were weaker than those in uninjured players. In both groups, those footballers with a restricted range of ankle dorsiflexion presented similar hamstring muscle strength to those with a normal range. Moreover, the poor and non-significant relationship between the hamstring strength and ankle dorsiflexion, as well as the low AUC value, indicates no effect of ankle joint mobility limitation on hamstring muscle strength. Based on the obtained results, it can be assumed that ankle dorsiflexion limitation, which is common in football players, is not a factor in weakening the hamstring muscle strength of either football players after ACL reconstruction or of those without injuries. However, some authors have reported that limited mobility of the ankle joint can have a destructive effect on the work of the lower limbs and may also be a factor increasing the risk of football injuries in its area [30,31]. Therefore, we have suggested that hamstring muscle weakness and increased risk of injury may occur due to factors other than limited ankle mobility. These observations may be of great importance in the selection of prevention methods by including a broad spectrum of physical techniques, not just exercises, that focus on the improvement of mobility or stability of the lower limbs.

## Figures and Tables

**Table 1:** Study group characteristics.

Outcome Measure	Group 1	Group 2	*p*
Number of subjects	24	23	
Age (years)	22.7 ± 3.6	22.5 ± 3.7	0.86
Body mass (kg)	77.3 ± 7.6	75.3 ± 9.3	0.88
Body height (cm)	175 ± 4	177 ± 3	0.95

*p—p* value.

**Table 2:** Comparison of strength variables for injured (Group 1) and right (Group 2) leg.

Outcome Measure		Group 1			Group 2				
	WBLT	Mean ± SD	*p* *	ES (d) *	Mean ± SD	*p* *	ES(d) *	*p* **	ES(d) **
Peak Torque/BW (%)	N	122 ± 23	0.98	0.03	160 ± 30	0.50	0.26	0.006	1.42
	R	121 ± 28			168 ± 31			0.0002	1.59
Average Power (W)	N	63 ± 19	0.50	0.29	88 ± 20	0.76	0.10	0.01	1.28
	R	58 ± 14			86 ± 18			0.0003	1.76
Total Work (J)	N	578 ± 114	0.26	0.55	610 ± 112	0.92	0.04	0.60	0.28
	R	517 ±106			605 ± 117			0.06	0.78

WBLT—Weight-Bearing Lunge Test; N—normal, R—restricted; *p* *—*p* value between subjects with normal and restricted ankle mobility within group; *p* **—*p* value between groups; ES *—effect size between subjects with normal and restricted ankle mobility within group; ES **—effect size between groups. Values are expressed as mean ± SD.

**Table 3:** Comparison of strength variables for uninjured (Group 1) and left (Group 2) leg.

Outcome Measure		Group 1			Group 2				
	WBLT	Mean ± SD	*p* *	ES (d) *	Mean±SD	*p* *	ES (d) *	*p* **	ES (d) **
Peak Torque/BW (%)	N	130 ± 17	0.94	0.05	161 ± 37	0.30	0.47	0.02	1.07
	R	131 ± 20			178 ± 35			0.0008	1.64
Average Power (W)	N	71 ± 19	0.74	0.17	86 ± 21	0.61	0.22	0.09	0.74
	R	68 ± 16			82 ± 14			0.01	0.93
Total Work (J)	N	648 ± 121	0.35	0.97	638 ± 123	0.80	0.10	0.80	0.08
	R	531 ±118			625 ± 119			0.10	0.79

WBLT—Weight-Bearing Lunge Test; N—normal, R—restricted; *p* *—*p* value between subjects with normal and restricted ankle mobility within group; *p* **—*p* value between groups; ES *—effect size between subjects with normal and restricted ankle mobility within group; ES **—effect size between groups. Values are expressed as mean ± SD.

**Table 4:** Correlation between WBLT and strength variables.

	Group 1				Group 2			
	WBLT (cm)				WBLT (cm)			
	Involved		Uninvolved		Right		Left	
	r	*p*	r	*p*	r	*p*	r	*p*
Peak Torque/BW (%)	−0.06	0.77	0.09	0.65	−0.18	0.40	−0.16	0.45
Average Power (W)	0.08	0.67	0.19	0.35	0.07	0.75	0.25	0.24
Total Work (J)	0.26	0.17	0.15	0.27	−0.07	0.75	0.05	0.79

WBLT—Weight-Bearing Lunge Test; r—Pearson’s correlation coefficient; *p*—*p* value.

**Table 5:** AUC for strength variables.

	Group 1				Group 2			
	Involved		Uninvolved		Right		Left	
	AUC	*p*	AUC	*p*	AUC	*p*	AUC	*p*
Peak Torque/BW (%)	0.481	0.87	0.50	0.97	0.59	0.43	0.59	0.45
Average Power (W)	0.45	0.71	0.52	0.83	0.48	0.92	0.41	0.48
Total Work (J)	0.40	0.42	0.65	0.16	0.50	0.95	0.47	0.81

*p*—*p* value; AUC—area under ROC curve.

## Data Availability

All data generated or analyzed during this study are included in this published article.

## References

[1] Wollin M., Thorborg K., Pizzari T. (2018). Monitoring the effect of football match congestion on hamstring strength and lower limb flexibility: Potential for secondary injury prevention?. Phys. Ther. Sport.

[2] van Dyk N., Farooq A., Bahr R., Witvrouw E. (2018). Hamstring and Ankle Flexibility Deficits Are Weak Risk Factors for Hamstring Injury in Professional Soccer Players: A Prospective Cohort Study of 438 Players including 78 Injuries. Am. J. Sports Med..

[3] Moreno-Pérez V., Rodas G., Peñaranda-Moraga M., López-Samanes Á., Romero-Rodríguez D., Aagaard P., Del Coso J. (2022). Effects of Football Training and Match-Play on Hamstring Muscle Strength and Passive Hip and Ankle Range of Motion during the Competitive Season. Int. J. Environ. Res. Public Health.

[4] Gabbe B.J., Bennell K.L., Finch C.F., Wajswelner H., Orchard J.W. (2006). Predictors of hamstring injury at the elite level of Australian football. Scand. J. Med. Sci. Sports.

[5] Biz C., Nicoletti P., Baldin G., Bragazzi N., Crimì A., Ruggieri P. (2021). Hamstring Strain Injury (HSI) Prevention in Professional and Semi-Professional Football Teams: A Systematic Review and Meta-Analysis. Int. J. Environ. Res. Public. Health.

[6] Moreno-Pérez V., Soler A., Ansa A., López-Samanes Á., Madruga-Parera M., Beato M., Romero-Rodríguez D. (2020). Acute and chronic effects of competition on ankle dorsiflexion ROM in professional football players. Eur. J. Sport Sci..

[7] Mason-Mackay A.R., Whatman C., Reid D. (2017). The effect of reduced ankle dorsiflexion on lower extremity mechanics during landing: A systematic review. J. Sci. Med. Sport.

[8] López-Valenciano A., Ayala F., Vera-García F.J., De Ste Croix M., Hernández-Sánchez S., Ruiz-Pérez I., Cejudo A., Santonja F. (2019). Comprehensive profile of hip, knee and ankle ranges of motion in professional football players. J. Sports Med. Phys. Fitness..

[9] Gonzalo-Skok O., Serna J., Rhea M.R., Marín P.J. (2015). Relationships between functional movement tests and performance tests in young elite male basketball players. Int. J. Sports Phys. Ther..

[10] Bahr R., Thorborg K., Ekstrand J. (2015). Evidence-based hamstring injury prevention is not adopted by the majority of Champions League or Norwegian Premier League football teams: The Nordic Hamstring survey. Br. J. Sports Med..

[11] Ekstrand J., Hägglund M., Waldén M. (2011). Epidemiology of Muscle Injuries in Professional Football (Soccer). Am. J. Sports Med..

[12] Bezodis N.E., Trewartha G., Salo A.I. (2015). Understanding the effect of touchdown distance and ankle joint kinematics on sprint acceleration performance through computer simulation. Sports Biomech..

[13] Morin J.-B., Gimenez P., Edouard P., Arnal P., Jiménez-Reyes P., Samozino P., Brughelli M., Mendiguchia J. (2015). Sprint Acceleration Mechanics: The Major Role of Hamstrings in Horizontal Force Production. Front. Physiol..

[14] Pattyn E., Verdonk P., Steyaert A., Vanden Bossche L., Van Den Broecke W., Thijs Y., Witvrouw E. (2011). Vastus medialis obliquus atrophy: Does it exist in patellofemoral pain syndrome?. Am. J. Sports Med..

[15] Almansoof H.S., Nuhmani S., Muaidi Q. (2023). Correlation of ankle dorsiflexion range of motion with lower-limb kinetic chain function and hop test performance in healthy male recreational athletes. PeerJ.

[16] Myers T.W. (2009). Anatomy Trains: Myofascial Meridians for Manual and Movement Therapists.

[17] Seymore K.D., Domire Z.J., DeVita P., Rider P.M., Kulas A.S. (2017). The effect of Nordic hamstring strength training on muscle architecture, stiffness, and strength. Eur. J. Appl. Physiol..

[18] Mizrahi J., Verbitsky O., Isakov E., Daily D. (2000). Effect on Running Kinematics. Hum. Mov. Sci..

[19] Waldén M., Atroshi I., Magnusson H., Wagner P., Hägglund M. (2012). Prevention of acute knee injuries in adolescent female football players: Cluster randomised controlled trial. BMJ.

[20] Timmins R.G., Bourne M.N., Shield A.J., Williams M.D., Lorenzen C., Opar D.A. (2016). Short biceps femoris fascicles and eccentric knee flexor weakness increase the risk of hamstring injury in elite football (soccer): A prospective cohort study. Br. J. Sports Med..

[21] Kim H.-J., Lee J.-H., Ahn S.-E., Park M.-J., Lee D.-H. (2016). Influence of Anterior Cruciate Ligament Tear on Thigh Muscle Strength and Hamstring-to-Quadriceps Ratio: A Meta-Analysis. PLoS ONE.

[22] Palmieri-Smith R.M., Lepley L.K. (2015). Quadriceps Strength Asymmetry After Anterior Cruciate Ligament Reconstruction Alters Knee Joint Biomechanics and Functional Performance at Time of Return to Activity. Am. J. Sports Med..

[23] Charlton P.C., Raysmith B., Wollin M., Rice S., Purdam C., Clark R.A., Drew M.K. (2018). Knee flexion strength is significantly reduced following competition in semi-professional Australian Rules football athletes: Implications for injury prevention programs. Phys. Ther. Sport.

[24] Powden C.J., Hoch J.M., Hoch M.C. (2015). Reliability and minimal detectable change of the weight-bearing lunge test: A systematic review. Man. Ther..

[25] Krause D.A., Cloud B.A., Forster L.A., Schrank J.A., Hollman J.H. (2011). Measurement of ankle dorsiflexion: A comparison of active and passive techniques in multiple positions. J. Sport Rehabil..

[26] Calatayud J., Martin F., Gargallo P., García-Redondo J., Colado J.C., Marín P.J. (2015). The validity and reliability of a new instrumented device for measuring ankle dorsiflexion range of motion. Int. J. Sports Phys. Ther..

[27] Banwell H.A., Uden H., Marshall N., Altmann C., Williams C.M. (2019). The iPhone Measure app level function as a measuring device for the weight bearing lunge test in adults: A reliability study. J. Foot Ankle Res..

[28] Larsson B., Karlsson S., Eriksson M., Gerdle B. (2003). Test–retest reliability of EMG and peak torque during repetitive maximum concentric knee extensions. J. Electromyogr. Kinesiol..

[29] Tiffreau V., Ledoux I., Eymard B., Thévenon A., Hogrel J.Y. (2007). Isokinetic muscle testing for weak patients suffering from neuromuscular disorders: A reliability study. Neuromuscul. Disord..

[30] Youdas J.W., McLean T.J., Krause D.A., Hollman J.H. (2009). Changes in active ankle dorsiflexion range of motion after acute inversion ankle sprain. J. Sport Rehabil..

[31] Wahlstedt C., Rasmussen-Barr E. (2015). Anterior cruciate ligament injury and ankle dorsiflexion. Knee Surg. Sports Traumatol. Arthrosc..

[32] Macrum E., Bell D.R., Boling M., Lewek M., Padua D. (2012). Effect of limiting ankle-dorsiflexion range of motion on lower extremity kinematics and muscle-activation patterns during a squat. J. Sport Rehabil..

[33] Mauntel T.C., Begalle R.L., Cram T.R., Frank B.S., Hirth C.J., Blackburn T., Padua D.A. (2013). The effects of lower extremity muscle activation and passive range of motion on single leg squat performance. J. Strength. Cond. Res..

[34] Howe L.P., Bampouras T.M., North J., Waldron M. (2019). Ankle dorsiflexion range of motion is associated with kinematic but not kinetic variables related to bilateral drop-landing performance at various drop heights. Hum. Mov. Sci..

[35] Lockie R.G., Callaghan S.J., Berry S.P., Cooke E.R.A., Jordan C.A., Luczo T.M., Jeffriess M.D. (2014). Relationship between unilateral jumping ability and asymmetry on multidirectional speed in team-sport athletes. J. Strength Cond. Res..

[36] Bennell K.L., Talbot R.C., Wajswelner H., Techovanich W., Kelly D.H., Hall A.J. (1998). Intra-rater and inter-rater reliability of a weight-bearing lunge measure of ankle dorsiflexion. Aust. J. Physiother..

[37] Oleksy Ł., Mika A., Sulowska-Daszyk I., Kielnar R., Dzięcioł-Anikiej Z., Zyznawska J., Adamska O., Stolarczyk A. (2023). The Evaluation of Asymmetry in Isokinetic and Electromyographic Activity (sEMG) of the Knee Flexor and Extensor Muscles in Football Players after ACL Rupture Reconstruction and in the Athletes following Mild Lower-Limb Injuries. J. Clin. Med..

